# Gleanings in Science and Medicine

**Published:** 1893-07-08

**Authors:** 


### Gleanings in Science and Medicine.

As the result of long experience, it is stated that the effects of imprisonment are far severer, bodily and mentally, on women than on men, so that equality of sentence does not necessarily carry with it equality of punishment.

Professor Rudolf Emmerich is said to have made an important discovery at Munich, namely that Asiatic cholera is a nitric acid poisoning produced by the Koch bacillus. Experiments made on guinea pigs and rabbits showed that nitric acid poisoning produced the same symptoms as those of cholera produced by inoculation. Professor Emmerich also points out that persons suffering from cholera show symptoms identical with those of nitric acid poisoning; and, it need hardly be said, that if these observations are well founded, a new chapter in the investigation of cholera has been opened.

A young girl of seventeen, named Harriett Walters, entered the employment of a japanner at Wolverhampton in January last. She earned about eight shillings a-week by brushing enamelled ware. To counteract the danger of inhaling poisonous lead fumes, a danger which was unavoidable in the work on which she was engaged, she wore a sponge or handkerchief over her mouth; and it appears that she was supplied with medicine and milk by her employers. But at the beginning of June she began to feel headaches and other symptoms of lead poisoning, and in a few days she died. The Government inspector reported that her employers bore a good character for looking after their employés' health; but the evidence laid before the coroner's inquest showed that the death was caused by chronic lead poisoning. Her daily work had thus killed her in less than six months.

Dr. P. J. Kolski's graduation thesis at the Moscow University deals with the effects of the varied meteorological conditions upon croupous pneumonia, a disease which has gained firm hold on Russian territory. The doctor's argument, which he deals with at great length, is definitely anti-microbic, and the writer maintains that weather changes, if they are not the primary cause, are yet responsible alone for the development of the malady. The Russian doctor's views of the case may be defined as exclusively meteorological, and his thesis, which is exhaustively worked out, is confined to a parallelism between the surrounding atmospheric variations and the etiology of the disease which he is dealing with. Dr. Kolski does not accept the old-established theory that high winds are productive of pneumonia, nor does he hold with Seibert's theory which considers that an absence of parallelism between the temperature curve and the curve of relative humidity is favourable to an appearance of the disease. The climatic conditions which, according to the writer's researches, have proved themselves most productive of cases of this disease, are when the temperature is lower than usual, and is uninterrupted with few diurnal changes; an abnormally high pressure of the barometer, and snow or rain during a northerly wind of moderate force. The years when strong and lasting winds have been registered are not those in which much croupous pneumonia was recorded.

A comprehensive plan for the transformation of Paris has been developed since 1848; slums have disappeared from Berlin since 1870; eighty-eight acres in the centre of Glasgow have been remodelled; Birmingham has transformed ninety-three acres of squalid slums into magnificent streets. Vienna has completed a stately outer ring round the centre of the city and is now beginning to remodel that. London is probably the most inconvenient, irregular and unmethodical collection of houses in the world.

A French contemporary publishes a curious incident in connection with the inmate of a certain lunatic asylum. We have often heard of remarkable gastronomic feats performed by the mentally afflicted, such as the swallowing of spoons and other articles of culinary use, but this is the story of the swallowing of a silver watch measuring 2½ in. in diameter. It appears that the perpetrator of this unprecedented deed was a man suffering from hallucinations, and temporarily confined in an asylum. Here, his wife had come to pay him a visit of the allotted length of time, and as her visit approached its close, the woman alluded to the fact, drawing out her watch to illustrate the same to her husband. Whereupon he became greatly excited, and in his frenzy seized the watch which she was holding in her hand, and swallowed it before she had time to check the action. Much alarm was felt as to the result of this deviation in the usual routine of customary diet, and the man was carefully attended to. The story concludes by adding that at the expiration of 14 days the watch reappeared *per naturalem viam*, and without any harm accruing to its consumer.

At twenty years of age a temperate person is supposed to have a chance of living for forty-four years. Should the same person, still living a temperate life, reach the age of sixty-six, the chances are that he will live fourteen years longer. At twenty years of age an intemperate person is calculated to have a chance of living only to the age of thirty-five; while if he survives to sixty, his chance of life is limited to eight years more. Vital statistics are, of course, subject to exceptions in individual cases; but facts such as these are most significant. They have been known for years, and, along with the equally well-known fact that crime and poverty are intimately associated with drunkenness, have done much to bring about the various proposals for regulating the sale of intoxicants. But, along with other medical aspects of the question (such as alcohol as a cause of disease), they are now seldom alluded to, owing to the fact that "temperance legislation" is now discussed almost exclusively from the political point of view. Nevertheless, in spite of the violent feelings excited by Veto Bills and proposals for the compulsory restraint of drunkards, the medical aspects of the drink question are of paramount importance; and the vital statistics bearing on the subject are worth remembering. A consideration of the high death-rate among innkeepers and licensed victuallers is not irrelevant; nor, in discussing the effect of alcohol on health, should we forget that the death-rate among the intemperate on beer is about 46 per 1,000, while that of the intemperate on spirits reaches the high average of 60 per 1,000.

